# Supplementary material for: Patterns of Intron Gain and Loss in Fungi
Source: PLoS Biol. 2004 Nov 30;2(12):e422. doi: 10.1371/journal.pbio.0020422 (PMC532390; doi:10.1371/journal.pbio.0020422)
Supplement: Table S1 — Also available at http://genes.mit.edu/NielsenEtAl/. (4.3 MB ZIP). [file pbio.0020422.st001.zip › NielsenEtAl/html/38.html]

AN3811.1.NCU04229.1.MG06945.1.FG10221.1


```
 CLUSTAL W (1.82) Multiple Sequence Alignments - Introns Inserted


Sequence 1: NCU04229.1	1146 aa
Sequence 2: FG10221.1	1023 aa
Sequence 3: MG06945.1	1103 aa
Sequence 4: AN3811.1	1016 aa
Alignment Length: 1238 aa
Number Identitical Residues: 392 aa
Alignment Score (without introns) 20323


MG06945.1 	----------MPAELVVIDSSE------SEGKGLGAD~RCSKTALGKRKAEP--------
NCU04229.1	----------MAENAIVLSDDDPTLSFDEDFRPQSSA~SATTTSLGKRKLS---------
FG10221.1 	-------MPLLQPEVIEILDDD-----DKASETGLNS~PTPSKSVQKRKHE---------
AN3811.1  	MVSTDDLDDLDTVTVCSFDDDTNSTPSEKDHDTTHAH1PSPEPESIKPKVKIPSPSPSRS
          	  ::..           : ..  : . ..           .     * * . .:.:.: :

MG06945.1 	---------VPPPIETIPWTDSEEEAEKER-------TEKKQAKGRRSAKKKKSIKSEPG
NCU04229.1	----------VSFEEKVDWSDDSDDGEK--------------LKARRNAKRRATSGSVRG
FG10221.1 	----------TEIEEKVEWTGDEDDLPT--------------KPKKRRGKRKSTR-----
AN3811.1  	TLASTHRIKSSPPDQKIPVDHATDGKSSDNDSFNEGEAVYKEFKNRKSRKRKRTSTAVAK
          	: :::   .     :.:      :   .. .: ....:  ..   ::  *:: :  :   

MG06945.1 	NAITNHGRRNARS--LAEAIRDLG-----------EDTAPEDNVHADIPEYLKHRKRQFN
NCU04229.1	RGSKGKGRGGKKT--AAATNRSRHDEIIELNQPTVRKEEETDYAEAFIPDYLLERRRKFN
FG10221.1 	------KKGGQDA--DAKKEVP-----------------EEDFEMADLPEYLIKRRRKFD
AN3811.1  	NKSSKAAAKDTFVGRVPRKKEVRGFRPTYLRDYKEEVLSDDDLMEDTLPDYLQKRRLQFD
          	   .     .   .  .              . .       *     :*:** .*: :*:

MG06945.1 	RDRATLQEAGLLLPPDYTDIDFTDDEDDGQRREVRPQFGDR--IKPCRPYEDVELERSAG
NCU04229.1	KDRELLRNAGLKLPPDYHDIYFSDDEK-GTRYEQRPKFEESSGIKPCRPYKDVELEYSAG
FG10221.1 	NKRKLHHDAALMIPPDYSGIHFEETGR-LRELEERPKFPESSGIKPSRPYKDIELPQSAG
AN3811.1  	RRLEHLQDAGLKLPPSYDHVYFSDDEQ----LDYLRRKPAFSGIKPCAEYKDISLPCSLG
          	.     ::*.* :**.*  : * :        :   :    :.***.  *:*:.*  * *

MG06945.1 	IIPAPIAQYLRDYQVDGVSFMHEAFVYQRGCILGDDMGLGKTVQVAAFLTAAFGKTGDFR
NCU04229.1	IIPACIAQYLRDYQVEGVKFLHQKFVYQRGCILGDDMGLGKTVQVAAFLTAAFGKTGDER
FG10221.1 	LIPASIAQYLRDYQVAGASFLHRKFVYQEGGILGDDMGLGKTVQVAAFLTVAFGKTGDER
AN3811.1  	VIPAPIAQWLRPYQVEGAAFLHQLFVYQKGGILGDDMGLGKTVQVIAFLTAAYGKTGDER
          	:*** ***:** *** *. *:*. ****.* ************** ****.*:***** *

MG06945.1 	DSKRMRKVKR-LGRRYY-KVLVVCPSSVMENWRQELARWGWWKVESYHGPGKDDVLDTAK
NCU04229.1	DAKRMRKMRR-AGDLWYPRVIIVCPGSLIQNWKNELDRWGWWHVDVFHGSNREDVLQAAK
FG10221.1 	DAKRLRQVRQ-YPDRWYPRILIICPGSLIMNWKNELDRWGWWHTDLFHGANKDDALSTAR
AN3811.1  	DDKRMRKMRRSEGDLWYPRTLIICPGTLIENWKSEMSRWGWWHFDAYQGSNKDLVLHAAK
          	* **:*:::::    :*.: :::**.::: **:.*: *****: : ::*..:: .* :*:

MG06945.1 	SGRLE~VMLTTPATYRLQKERVNTVSWDAVVVDECHNIKERRSEITKAMTEVNALCRIGL
NCU04229.1	SGRIE0NTCVS--------------------------------ETTRAMDKINAMCRIGL
FG10221.1 	AGRVE~IMITTYDTYKNSRSAINLVQWDAVIADECHRLKDRSSETTKALNEINALCRIGL
AN3811.1  	SGRVE~ILITTYSTYLKNRDAVNMVDWDCVIADECHAIKERSSETTKAMNDINALCRIGL
          	:**:*    .:  :   . .  .  . ..   ...   ..  :* *:*: .:**:*****

MG06945.1 	TGTAIQNKYEELWTLLNWTNPGRFGTFGEWKTSISRPLTLGQSHNATLQELSMARKTAKK
NCU04229.1	TGTAIQNRYEELWTLLNWTNPGYFGTRAEWNESITKPLTAGQSHDATLKQLSIARTTAKK
FG10221.1 	TGTAIQNRYEELWTLLDWTNPGHFGTLPEWTQRVTKPLTVGQSHDATVAQLSLARTTADK
AN3811.1  	TGTAIQNRYEELWTLLNWTNPGRMGPVTTWKSHVSEPLKIGQSHDATVQQLRRARVTAKK
          	*******:********:***** :*.   *.  ::.**. ****:**: :*  ** **.*

MG06945.1 	LVQNLLPDYFLRRMKSLIAHQLPKKRDRVVFCPLTDLQTTAYERFIASEEVELVRRSHEL
NCU04229.1	LVQNLLPEFFLRRMKSLIAHQLPKKSDKVVFCPLTDVQRDAYENFLEGEHVTFILNAYQP
FG10221.1 	LVHNLLPQYFLRRMKSIIANQLPKKTDRVVFCPLTDFQRDAYENFLSSADIEILRTLSEP
AN3811.1  	LVENLLPQFFLRRMKTLIADQLPKKIDRVVFCPLTDTQAQAYERLLDSDIIHYIKTSTNK
          	**.****::******::**.***** *:******** *  ***.:: .  :  :    : 

MG06945.1 	CDCGSGEKRGHCHYKHLEDGRTWNMLVFPCILTLQNLANHLTLIVPKIGEPGDKAEKSLA
NCU04229.1	CKCHSGRAGGFCCYKTLSDGRTWKSYVFPSIITLQKIANHLTLLIPSSSDPKEKQRSELN
FG10221.1 	--CCGGKKKGWCCNSLLPDGRRWQNIVFPSMMVLQKLANHLTLLVPHTTDLEAKHETDIK
AN3811.1  	CDCGSGRKAGWCCHQYLPSGRRWQTYVFPAMQILQKLSNHLAILIPQSTDSFEKQEKDKE
          	..* .*.  * *  . * .** *:  ***.:  **:::***::::*   :   * ...  

MG06945.1 	RLRICVPDKWEQLYKLRDSPLIQMDPEFCGKWRVLKDLLKFWEGNGDKVLVFSHSVKLLQ
NCU04229.1	VLQTCAPDTWKELYNNRESMLSLANPEFCGKWKILRKLLRFWHESGDKVLVFSHSVRLLR
FG10221.1 	TLQTCLPDTWKYVYDNRDRIKNLVNPEFCGKWKVLKKLLKFWHSNGDKVLVFSHSVRLLR
AN3811.1  	MLEIAVPDEWERLYRTRDSIINYADAEYCGKWKVLRKLLKWWHGNGDKVLVFSHSVRLLK
          	 *. . ** *: :*  *:      :.*:****::*:.**::*. .***********:**:

MG06945.1 	VLRALFQTTHYSYSYLDGSLAIEERQQIVDDFNSDPQQFVFLISTKAGGVGLNITSANKV
NCU04229.1	ILQHLFHNTSYNVSFLDGALSYEERQRVVDEFNTDPRQFVFLISTKAGGVGLNITSANKV
FG10221.1 	ILHHLFTNTSYTVSYLDGSLSYEVRQEVVDTFNSDPTQFVFLISTKAGGVGLNITSANKV
AN3811.1  	MLQMLFNHTSYNVSYLDGSMTYETRAKVVDEFNSDPRQFVFLISTRAGGVGLNITSANKV
          	:*: **  * *. *:***::: * * .:** **:** ********:**************

MG06945.1 	VIFDPHWNPSWDLQAQDRAYRIGQ0TRDVDVFRLVSQGTIEEIVYARQIYKQQQANIGYN
NCU04229.1	VIFDPHWNPSYDLQAQDRAYRIGQ~IRDVDVFRLVSAGTIEEIVYARQIYKQQQANIGYN
FG10221.1 	VIIDPHWNPSYDLQAQDRAYRIGQ~TRDVEVFRLISLGTVEEIVYARQIYKQQQANIGYT
AN3811.1  	VIVDPNWNPSHDLQAQDRAYRIGQ~TRNVEVFRLISAGTIEEIVYARQIYKQQQANIGYN
          	**.**:**** *************  *:*:****:* **:*******************.

MG06945.1 	ASNER2RYFTGVQQDNSRKGEIFGLENIFTFRPDRVVLRDIVNKTNVAEAKVGVYLQDID
NCU04229.1	ASNER~RYFKGVQRDKNRKGELFGLENLFTFHADQVVLRDIVNKTNIAEAKAGVNLTDID
FG10221.1 	ASSER~RYFKGVQQDTDRKGEIFGLANIFLYHNDSGLLQDIVNKTNIAEAKAGVHLVDVD
AN3811.1  	ASSER~RYFKGVQEKKDQKGEIFGLQNIFEYQNDNIVLRDIVNKTNIAESKAGVQVMDIE
          	**.** ***.***....:***:*** *:* :: *  :*:*******:**:*.** : *::

MG06945.1 	IESAIKDNEEAGIPIKLEPGSSGDAEERGASQLADFLTSKPQVKPGGENHERS---PGHD
NCU04229.1	MEKAVKDEDEELDFIKKE--SEDKDDDTGMSSLAKLVTAEEPDKLLEASKSKK---PKSD
FG10221.1 	MEKAAKDE-ENLGIIKNE--APDVED-GGMSQLASLLTSENQQRMLDSKKAKK---PKSD
AN3811.1  	LELEEAGSEDGSWSRNPD------DDDEAMSQLAARIRGETDDEVIFPHRQQSTTPSKHD
          	:*    ...:     : :       :. . *.**  : .:   .     : :.::..  *

MG06945.1 	AIQAILSSAGVGYTHDNAEVVGTSKVEAQLSRKAERDHEIDEK----EALKVLFADSQDV
NCU04229.1	AIAAILASAGVEYTHENSEVIGTSKVEAQLSRRAELAANAADSQLFPGANNALFADSTES
FG10221.1 	AIQAILTSAGVEYTHDNSEVIGSSKIEEQLSRRAAMASYSTQ-----EGQSALFADPG--
AN3811.1  	PIQAILAGAGVEYTHLNNEVVGSSKVEEQLSRQAQLAEDDSL------ANLQVFCDSQ--
          	.* ***:.*** *** * **:*:**:* ****:*              .   :*.*.   

MG06945.1 	GARPLYEHDQEAWDVDEMGEGDAWQSKRPETWFRPRLRYNPPEEVRLRQFCSMAKEFSFS
NCU04229.1	------SDGGDDDDDDEDQED--LNGPARKKMLRMHYKFNPPEDVKRRQFCSMAREFGFQ
FG10221.1 	---------------DERGEG--LFG-----------IYNPPEEAKLRQFCEMAKEFGFA
AN3811.1  	----------------DEVAGAVVAPTFQKNGRPVTFKYHPPEDVMKRQFCSMARRFGFP
          	                :   .        ..       ::***:.  ****.**:.*.* 

MG06945.1 	NAIEFALVVESWTQEQRRNCLDMFYKQRESELLKTTLAG-EAKKLDKHEPEVKTESTSAR
NCU04229.1	NATDFALVVESWTQEQRRNCLDTFYRRREVKLLEQEMAKMEAEKETEDDDDENDESTMVK
FG10221.1 	NATEFALVVESWTQEARRNCLDLFYKKREAKLIQEGIIKEEDVKDVAKDETHDPDAKEEV
AN3811.1  	NATEFALVVESMTQAERRACLDRWYRERRQALLE--------------------------
          	** :******* **  ** *** :*:.*.  *::                          

MG06945.1 	TKT----EVPEEHGMVGLENVQTKSKV---------------------------------
NCU04229.1	TDDDDDDETVDLEKAVANEEILVLGESKPNAGVEVTRGEIAEKRAVAGAAERGEEGDPLR
FG10221.1 	KDD-----KMEISSALKDEDIKDNKEN---------------------------------
AN3811.1  	------------------------------------------------------------
          	                                                            

MG06945.1 	-----------------------QIKNEAGVADSKVKMEPVNDEPESDSDDLDAVKVKDE
NCU04229.1	GTGALGYGVRSLPNARAGASASPVPKAEAGERRTDETATIVSEAGKPVDSGASSVAVKTE
FG10221.1 	-----------------------VLKREN--IKTEMISTKVEEEKK----------VKTE
AN3811.1  	------------------------------------------------------------
          	                                                            

MG06945.1 	DLSKIKYEPGLATIKRQ----------SSIFFFDDDDEDDEL
NCU04229.1	GVEEPRTVTVGAGTSFDNTAAAGQKKVTTIFLYDDEDEDDEL
FG10221.1 	G----------AGSSIR----------TSIFLSDDDD-DDEL
AN3811.1  	------------------------------------------
          	
```
